# The War in Ukraine and Migration to Poland: Outlook and Challenges

**DOI:** 10.1007/s10272-022-1053-6

**Published:** 2022-06-07

**Authors:** Maciej Duszczyk, Paweł Kaczmarczyk

**Affiliations:** Centre of Migration Research, University of Warsaw, Pasteura street 7, 02-093 Poland, Poland

**Keywords:** F22, O15, R23

## Abstract

The war initiated by Russia against Ukraine in February 2022 has resulted in the largest refugee migration in Europe since World War II, estimated by UNHCR (2022) at 6.3 million persons. In the first two months, almost 3.5 million war refugees crossed the Polish border, of which over 95% were Ukrainian citizens.

The outbreak of war in Ukraine has impacted many spheres of political, economic and social life. In particular, the flight from war zones drastically changed the migratory situation in Ukraine itself as well as in many countries of the EU, including Poland. Poland is playing the most important role among the countries receiving war refugees from Ukraine,[1] with around 3.5 million persons who arrived in Poland between 24 February and mid-May 2022. As we show in this article, this phenomenon is due not only to geographic factors (common border) but also due to the long-lasting tradition of (labour) migration between Ukraine in Poland. This notwithstanding, the unprecedented inflow of war refugees clearly raises questions about future developments and challenges related to the presence of Ukrainian citizens in Poland. This contribution presents an attempt at estimating the possible future stocks of immigrants from Ukraine in the country and points out related challenges. For obvious reasons, this attempt is subject to great uncertainty. However, the presented scenarios indicate that regardless of developments on the front line, we have to reckon with the fact that the number of immigrants from Ukraine in Poland will be significantly higher in the coming months (or maybe years) than at the beginning of 2022, and this poses certain challenges for public services and public institutions in Poland. We show that labour immigration to Poland, the crisis on the Polish-Belarusian border, and an influx of war refugees from Ukraine changed the status of Poland from a typical emigration country to an immigration one, without going through the intermediate phase, i.e. the emigration-immigration status. On top of that, in the second half of 2021 and the first half of 2022, Poland and refugees were a focus not only in the media but also in political discussions at the highest levels. This will have, both in the medium and long term, a huge impact on the perception of Poland in the world and could be (and possibly will be) a subject of internal political debate.

The first section of this paper discusses the contextual issues that, in our understanding, explain the patterns of recent inflows and allow for the understanding of the reception practices. The second section presents the process itself and provides the first estimates of future stocks of Ukrainian immigrants in Poland. In the concluding section, we look at the most important challenges faced by Poland as a destination country and by war refugees themselves.

## Contextual issues

The recent inflow of Ukrainians fleeing the war zones to Poland is, by all means, an unprecedented event. This section examines several contextual factors that, firstly, explain, to an extent, why Poland is the major destination country and, secondly, help predict and understand the development of Ukrainian migration and the presence of Ukrainians in Polish society in the future. It is important to note that, just a decade ago, Poland was not an immigration country. On the contrary, due to the post-2004 mass mobility to the West, the migration balance of the country was negative (Górny et al., 2010; Okólski, 2012; King and Okólski, 2018). Particularly if long-term immigration is considered, the inflow of immigrants to Poland was very limited. According to the 2011 population census, the total number of foreigners staying in the country was estimated at approximately 100,000. Just seven years ago (in 2015), Poland was ranked as one of the last among EU member states in terms of the share of immigrants in the total population. Immigration to Poland also had several important qualitative features, starting with a very limited number of source countries (with a clear majority of post-Soviet countries and Ukraine as the most important country of origin), through a clear concentration in a few big agglomerations (with Warsaw and the Mazowieckie region playing a central role). It also had predominantly tem-

1 We use the term "war refugees from Ukraine" so as to adequately reflect their specificity. These people are not granted refugee status under the 1951 Geneva Convention, and most of them also do not intend to apply for one of the forms of international protection. Therefore, it was necessary to find another term for their status in the European Union. The proposed term includes very different categories of people, both Ukrainian citizens and foreigners who, at the outbreak of the war, were on the territory of Ukraine and left it by crossing the border with, for example, Poland. Thus, citizenship does not matter here, but only the fact of leaving Ukraine after 24 February. In this article, we use the term "war refugees from Ukraine", but unless otherwise indicated, we only refer to Ukrainian citizens.

© The Author(s) 2022. Open Access: This article is distributed under the terms of the Creative Commons Attribution 4.0 International License (https://creativecommons.org/licenses/by/4.0/).

Open Access funding provided by ZBW – Leibniz Information Centre for Economics.

**Maciej Duszczyk**, University of Warsaw, Poland.

**Paweł Kaczmarczyk**, University of Warsaw, Poland.

porary or even circular mobility (Górny and Kaczmarczyk, 2019; Górny et al., 2010).

This picture changed substantially after 2014, i.e. after the first war in the eastern part of Ukraine. In a very short time, Poland became a European leader in newly issued residence permits and even a global leader when the seasonal foreign workforce was taken into account. According to available estimates, the stock of immigrants in Poland has increased from around 100,000 in 2011 to more than two million in 2019 (Statistics Poland, 2020). Ukrainian citizens represented the majority of this population, and certainly, the radical change in the migration situation in Poland is attributable to the inflow from this country. This was possible only because of an interplay of factors operating on two sides of the process (Górny and Kaczmarczyk, 2018, 2019; Górny, 2017). First of all, the 2014 Russian invasion of eastern Ukraine and the related socio-economic developments in Ukraine created a large migration potential. Nonetheless, the "transformation" of Poland into a country of immigration occurred due to its fast economic growth and related, persistent (and growing) demand for labour in the Polish labour market. As a result, and contrary to expectations of some observers, the potential mentioned above did not transform into humanitarian migration. Rather, Poland experienced a mass increase in labour migration, particularly based on a simplified procedure, which made Poland one of the most liberal regimes in terms of the employment of foreigners (additionally, being a fast-growing recruitment sector also contributed to the increase in the scale of labour migration).

There are several structural characteristics of pre-2022 war migration from Ukraine to Poland that are highly relevant in the context of recent inflows:

- Already before the war, there was a substantial group of Ukrainians working/residing in Poland that can be estimated at around 1.35 million (based on the Central Statistical Office's data); this group was mostly male and comprised of predominantly economically active persons (over 95% of the total).

- Ukrainian immigrants clearly dominated in the case of all possible channels of inflow into the Polish labour market; if we consider three major channels of inflow over the period 2018-2021, Ukrainians made up 88% of declarations, 71% of work permits and 98% of seasonal work permits.

- Immigration – including migration from Ukraine – has been more equally distributed across the country than in the pre-2014 period, with immigrants being present in almost all regions of Poland (with a few important concentration centres – big agglomerations).

- Due to specific forms of migration, i.e. temporary or even circular mobility between Ukraine and Poland, the two countries were connected through well-developed transportation routes (and Poland and the Polish labour market could be described in terms of well-trodden social and economic spaces).

- As in many similar cases, such a massive migration was possible not only due to the active involvement of formal and informal recruiters but also because it was strongly driven by well-developed migrant networks (Kindler and Wójcikowska-Baniak, 2019).

- Despite the increasingly common presence of Ukrainians in the Polish labour market, the scale of economic and social/cultural tensions remained at relatively low levels. This can be attributed mainly to very positive developments in Poland's economy and a flourishing labour market, with the lowest levels of unemployment recorded since the systemic change in 1989 (Duszczyk and Matuszczyk, 2018).

It is important to note also that the substantial inflow of immigrants to Poland took place in practice without a coherent and (clearly) articulated migration policy. In 2009, the Polish government attempted to create a coherent strategic document. Such a document was accepted in 2012, but it has been rejected by the government that came to power in 2015, without replacing it with a new one (until today). It can be concluded that the migration policy pursued in Poland is highly dispersed and continuously focuses on liberalising access to the labour market. As a consequence, before the 2022 war and the massive inflow of war refugees from Ukraine, there was no general integration policy in Poland (except measures dedicated to refugees, but this group constituted a very small share in the total number of persons arriving in Poland).

## Ukrainian migration to Poland after the Russian invasion

The war initiated by Russia against Ukraine in February 2022 has resulted in the largest refugee migration in Europe since World War II, estimated by UNHCR (2022) at 6.3 million persons. In the first two months, almost 3.5 million war refugees crossed the Polish border, of which over 95% were Ukrainian citizens. Figure 1 presents the scale of the border traffic between Ukraine and Poland and points to a remarkable increase in the scale of mobility in the first two to three weeks after the outbreak of the war.

Figure 1 shows that most of the inflow is the consequence of the first few weeks since the war started. The cumulative outcome of this migration is over 3.46 million inflows

Figure 1
**Daily border traffic between Ukraine and Poland, 24 February - 19 May 2022**
in thousands

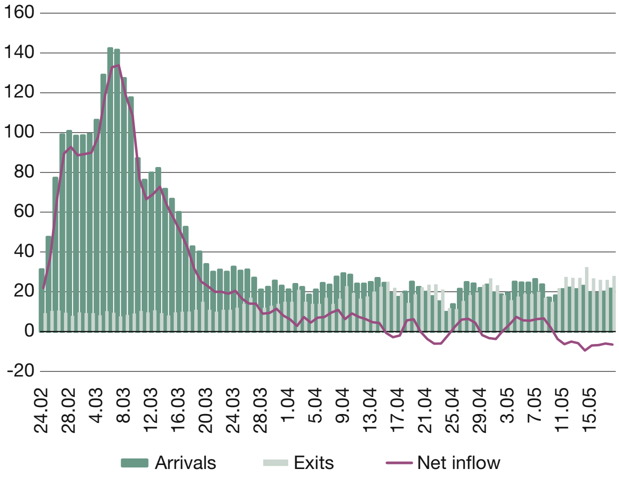

Note: Including non-Ukrainian citizens.

Source: Own elaboration based on the Border Guard data.

Figure 2
**Weekly changes in daily border traffic between Ukraine and Poland, 24 February - 19 May 2022**
in %

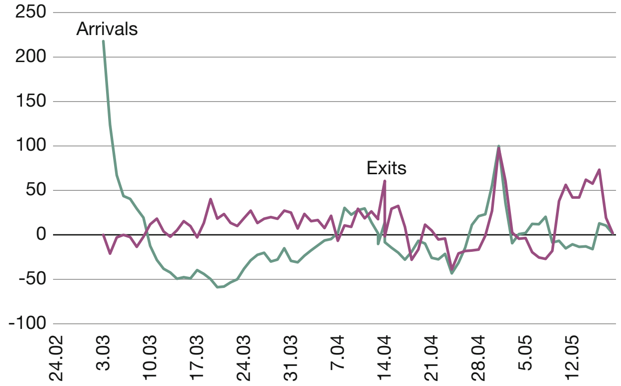

Note: Including non-Ukrainian citizens.

Source: Own elaboration based on the Border Guard data.

and over 1.39 million outflows. Figure 2 presents the weekly changes in the number of inflows and outflows and shows that since early May, there is an apparent tendency to move out of Poland rather than to leave Ukraine and arrive in Poland. Additionally, the volatility of both inflows and outflows is very high, which reflects changing war-related realities, but also points to the fact that we are dealing with a highly mobile population that is inherently interested in returning to their places of origin (if only possible). This mobility pattern resembles to some extent the reality of pre-war migration between Ukraine and Poland, which comprised large numbers of temporary migrants and circulants moving between the two countries regularly. We still lack data to estimate the scale of the phenomenon, but anecdotal evidence shows that some of the labour migrants continue their trips despite the war.

The above-mentioned numbers (almost 3.5 million arrivals in Poland) equalled more than 60% of all border crossings with Ukraine's neighbours. At the same time, more than 1.3 million people left for Ukraine during the period under consideration. Among them, there were about 50,000-60,000 people who had lived in Poland before the outbreak of war and returned to Ukraine to join the army or territorial defence. This means that the net flows of war refugees crossing the border were about 2.2 million. It does not mean, however, that so many people arriving in Poland were still in the country at the end of April 2022. Those who only passed through Poland on their way to other countries, especially the European Union and – to a much lesser extent – Canada, the USA or Israel, should be subtracted from this total flow. Based on available register data from receiving countries, this number can be estimated at 800,000 people.[2] At the same time, we also dealt with arrivals of war refugees to Poland from Ukraine who, after a short stay in other countries (mainly EU countries), decided to move to the country which is relatively close in cultural and linguistic terms (leaving aside the natural tendency for staying as close to the border with Ukraine as possible). Their number can be estimated at 70,000-80,000.[3] Summing up, the number of war refugees who were staying in Poland at the end of April 2022 can be estimated at 1.4-1.55 million people (we will use the latter estimate for further assessments).

The influx of war refugees from Ukraine has one very important feature. Those crossing the Ukrainian border with EU member states and Moldova are immediately covered by the provisions of the Temporary Protection Directive,[4] which grants them numerous rights. In principle, it makes the status of war refugees from Ukraine similar, but not the same, as that of EU citizens in terms of rights under the free

2 This number was obtained by adding the numbers (presented in official statistics or during government press conferences) from countries not bordering with Ukraine.
3 This number was cited by the Polish government when the law on help for Ukrainian citizens of 12 March 2022 was amended.
4 Council Directive 2001/55/EC of 20 July 2001 on minimum standards for giving temporary protection in the event of a mass influx of displaced persons and on measures promoting a balance of efforts between member states in receiving such persons and bearing the consequences thereof.

Table 1

**Demographic structure of war refugees who registered to obtain the PESEL number in Poland**

| | Number of war refugees | Share (%) of total |
|---|---|---|
| Children (aged 0-18) | 519,567 | 47.35 |
| Working age | 503,071 | 45.85 |
| Female | 460,361 | 41.96 |
| Male | 42,710 | 3.89 |
| Retirement age | 74,579 | 6.80 |
| Female | 63,878 | 5.82 |
| Male | 10,701 | 0.98 |
| Total | 1,097,217 | 100.00 |

Note: Data as of 15 May 2022. PESEL is a personal identification number.

Source: Own elaboration based on the PESEL register.

movement of persons. This is the first time in EU history that the Directive's provisions have been put into practice.

The estimations based on border crossing data can be supplemented with early information on the registration process, as Ukrainians are expected to register to obtain the Polish ID number (PESEL), necessary to get access to public goods and services. As of 15 May, the number of registered persons was as high as 1.1 million (see Table 1), with a very specific demographic structure: over 47% children (persons aged 0-18), with a majority of them (34% of the total) aged 3-14, 42% females (at working age) and roughly 7% elderly (persons at retirement age, defined as 60+ for females and 65+ for males). Places of registration clearly reflect the locations of the biggest Polish agglomerations, with Mazowieckie (20% of the total), Śląskie (10%) and Dolnośląskie (10%) playing the most important role. The Polish Ministry of Family and Social Policy reports that so far around 160,000 adult war refugees have already entered the Polish labour market (Business Insider Polska, 2022).

Other data is scarce and very incomplete. For this reason, we refer only to the data provided by the city council of Warsaw (as the city that has received the largest number of war refugees). According to the available data, the number of persons who arrived in Warsaw was as high as 700,000 (as of 24 April 2022). Out of these, approximately 300,000 persons were still staying in the city, and around 100,000 registered to obtain their ID number. The majority of persons who arrived in Warsaw have found shelter in private flats/homes, and the number of persons in temporary housing was as high as 90,000 (cumulated number). The scale of the challenge faced by the local administration is visible through the number of children staying in Warsaw, estimated at 150,000, which massively increased the number of children of school age in the city. Out of those, roughly 16,500 have already been integrated into the schooling system (data courtesy of Warsaw City Council).

## Outlook

We are perfectly aware of how difficult it is to predict the future of Ukrainian war refugees in Poland (and other countries). Their numbers – as well as the number of those returning to Ukraine – mainly depend on war-related developments and the future reconstruction of the country. Additionally, one may select other criteria defining particular scenarios (e.g. EU policy, socio-economic situation in Poland, attitudes towards war refugees). The main aim of the exercise provided in this section is to estimate the scale of possible challenges Poland will expectedly face.

We estimate the scale of Ukrainian presence in Poland in April 2022 at approximately 2.9 million. Importantly, this number is a sum of two sub-populations: those persons who were staying in Poland before the war (around 1.35 million) and those who arrived since then (around 1.55 million, as discussed above). This is a key remark, as our further estimates refer not only to the recent inflows but also to those persons who were/are unable to return to Ukraine because of the war and, as a consequence, are also experiencing the impact of the situation in Ukraine. Due to the very special demographic structure of the newly arrived war refugees, we estimate that the total population is drastically different from typical labour migration as observed before the war, i.e. 40% women (aged 18-65), 26% children and 2% elderly.

In the next step, we consider three main hypothetical scenarios (numerical estimates are presented in Figure 3 – please note that these are rough estimates only based on certain assumptions concerning the main demographic groups). In all cases, we consider the short/medium term, i.e. we are estimating the stock of immigrants from Ukraine in the next 12-20 months, i.e. until the end of 2023.

In the first scenario (long continuous war, mostly on a regional level), we expect the continuation of the conflict (with varying intensity, scope and scale of activities) for the next several years, i.e. similar to the war over Donbas and Luhansk after the Russian aggression of 2014. This would mean that within the next 18 months, (large) parts of Ukraine will still be under threat. This will result in a continuous flow of refugees, but also economic migrants to Poland. There will certainly also be numerous temporary and permanent returns to regions not affected by the war, mainly western Ukraine. It should be assumed that, as a result of the continuation of the conflict, which will have different phases of calming down and intensifica-

Figure 3
**The future stock of Ukrainian immigrants in Poland, possible scenarios**
in millions

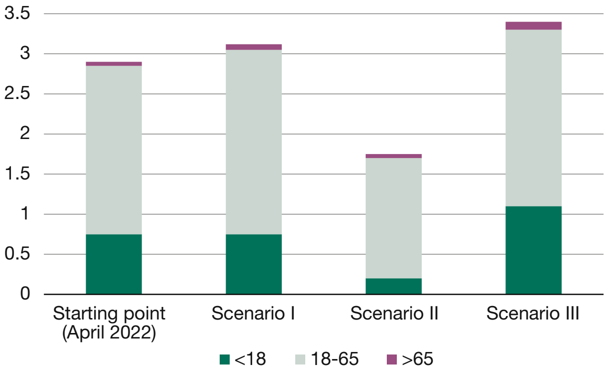

Note: Scenario I is a long continuous war, mostly on a regional level; Scenario II is a quick and lasting peace; Scenario III is similar to Scenario II, but it is assumed that the war will lead to greater destruction also in the west of Ukraine, but a peace agreement will be signed earlier than assumed for Scenario I.

Source: Own elaboration.

tion of fighting, the economic situation in Ukraine will be bad, which will stimulate more intensive economic migration than in the past. This means that the structure of the inflow may change, with an increasing share of men and older people (we assume that the ban on leaving Ukraine by men aged 18-60 will be significantly liberalised or even lifted). Presuming factors influencing flows and patterns of residence of particular demographic groups are as described above, this scenario implies that about 3.1 million Ukrainians will reside in Poland in the medium term (economic migrants who came to Poland before outbreaks of the war and war refugees). Considering the assumptions described above, we can conclude that the demographic structure will be as follows: 24% children, about 37% women and 37% men (at working age). About 72% of people will be of working age, which means that the age structure would be similar to the one we have now.

In the second scenario (a quick and lasting peace), one should assume a quick (by autumn) conclusion of peace, which will stabilise the situation in the short run and will also bring relatively favourable conditions for Ukraine (territorial, reparations, possibilities of joining the EU, etc.) in the medium and long term. It would mean a relatively large reduction (during the 12 months following the signing of the peace agreement) in the stock of women and children, some outflow of men (including those residing in Poland in the pre-war period), and a stable stock of elderly people. In this scenario, it should be assumed that the number of Ukrainian citizens staying in Poland will stabilise at around 1.75 million, of which 1-1.25 million would be "pre-war" immigrants (mainly males) and 0.5-0.75 million war refugees would transform into "post-war" immigrants (mainly females, children and the elderly, to a large extent family members of those staying in Poland before the war). It should be assumed that mainly people from eastern Ukraine will stay in Poland, since the destruction of the infrastructure is the greatest there, and reconstruction will take the longest. The demographic structure would be as follows: 11% children, about 37% women and about 49% men. The economically active adult population would account for about 86%, which would mean a gradual but rather slow return to the structure of the population residing in Poland before the outbreak of war (share of economically active persons: over 95%).

The third scenario is – at the level of assumptions – similar to the previous one, but we assume that the war will also lead to greater destruction in western Ukraine, whereas a peace agreement will be signed earlier than assumed in scenario one. This means that regardless of the conditions of the assumed peace, an additional influx of children, the elderly and women should be expected, as well as a possible outflow of men (ongoing fighting, reconstruction of the country after signing the peace agreement). In this variant, one should assume an increase in the number of Ukrainians in Poland to around 3.4 million (by the end of 2023). This is due to the potential devastation caused by the prolonged war and the partial integration of Ukrainians into Polish society, which would encourage part of the population to remain in Poland for longer. The demographic structure would be as follows: 32% children, 40% women and about 25% men. The economically active adult population would account for about 65%, due to the increased proportion of minors compared to the pre-war (or even baseline) period.

Scenarios two and three should assume significant investment to rebuild damaged infrastructure, financed either by international aid or reparations. If the funds for this purpose are substantial, it may cause an exodus of workers currently employed in the construction industry in Poland. However, it is difficult to assume that Ukraine's GDP will quickly return to pre-war values. Therefore, labour immigration to Poland and other EU countries will be higher than before the war (with higher shares of females). There will also be a reunification of families that are now separated, especially from areas where Ukrainian control will not be restored or bordering them, as well as those most damaged by war. The end of the war refugee/humanitarian immigration should be assumed in this scenario. The period of temporary protection in the EU, granted based on the 2001 Temporary Protection Directive, is likely to end. An EU Council decision will be required. Numerous actions by the Ukrainian government to induce emigrants to return can also be expected.

In another scenario, which is currently unlikely and unwelcome but cannot be completely ruled out, Russia gains military advantage and eventually occupies much of Ukraine's territory. Ukrainian citizens, knowing what happened in Bucha, Mariupol and other cities/territories occupied by Russia, flee en masse to Poland and other European countries. In such a scenario, the number of refugees could even exceed ten million, of which about 60% would stay for some time in Poland. In this scenario, all existing assumptions would have to be changed. Poland and the European Union would be hit by a humanitarian crisis that would require a massive relocation within the EU. It would be crucial to provide basic needs in the form of housing, food, medical care, etc. However, this scenario is not explored further within this article.

As shown in Figure 3, all three scenarios considered would mean a substantial increase in the number of Ukrainian citizens residing in Poland as compared to the pre-war situation, however, the scale of this presence varies depending on the scenario. Differences lie not only in the scale of the process but also in its structural features with scenarios one and two linked to a significantly higher presence of children (and elderly).

## Challenges ahead

Long-term stays of war refugees in Poland, depending on the presented scenario, will generate numerous challenges in the field of social services, which must be prepared to serve a larger number of people. This is the issue we discuss in this final section.

We believe that in each scenario, the key challenge is to provide housing infrastructure. It is unsustainable (even in the short term) for war refugees to live mainly in private houses or apartments and this has become a common "reception practice" in the first weeks of the war. While the challenge is easier overcome in the summer months, by autumn it will be an absolute priority. The solution to the situation would be relocation within the EU, within Poland, and the construction of modular housing estates, in which people who do not have an apartment would be able to spend autumn and winter. In the absence of immediate actions or the case of an additional influx of war refugees, it may be necessary to build large reception centres (as in the first phase of the inflow) or centres of temporary stay.

In scenarios one and three, it will be a massive challenge to provide education and care to children from Ukraine residing in Poland. In an extreme situation, there could be as many as one million children in need of care and education. Without it, it is difficult to expect most mothers or family members performing care functions to be able to take up employment. Therefore, it is necessary to prepare extraordinary solutions based on a few models (in fact, all of the models are already functioning – to a limited extent of course). In the first one, Ukrainian children will continue to follow the Ukrainian curriculum, and the goal of the government and local governments will be, on the one hand, to provide infrastructure for distant learning and, on the other, to recognise the qualifications of Ukrainian teachers residing in Poland and to create Ukrainian school classes, particularly in big cities. In the second model, preparatory classes can be created to help Ukrainian children get ready for entry into Polish schools next year. In the third model, directed only at those children from Ukraine who have a sufficient command of the Polish language, the possibility of attending Polish schools on the same terms as Polish children should be created. The decision to choose a given model should be left to parents (but be conditional on the command of the Polish language).

All three scenarios described in Figure 3 show that the presence of elderly war refugees, who presumably require regular medical care, is limited. However, even if numbers of elderly are not high, this is a completely new phenomenon in Poland as before the war as many as 97% of the total stock of immigrants constituted persons of working age. Furthermore, the Polish health system has been strongly affected by the pandemic and many people have postponed their medical care. Meanwhile, an additional one to two million people are now entitled to use the health care system, and this will surely create severe challenges (both in terms of general and specialist health care). Considering additional risks resulting from the COVID-19 pandemic, it may be necessary to ask other member states for support to provide temporary hospitals and to post doctors in Poland for a certain period. Solving communication problems will also be crucial.

As we showed in the first part of this text, the presence of Ukrainians in the Polish labour market was significant already before the war. It can therefore be assumed that there should not be a problem with employing another several hundred thousand people (and this is already partially confirmed by the fact that almost 150,000 newly arrived war refugees have entered the Polish labour market). Unfortunately, such a perspective can be too optimistic. As mentioned in the previous section, the recent inflow comprises mainly women with children, while before the war, Ukrainians had been employed in Poland mostly in male-dominated occupations. Thus, we may be dealing with mismatches in terms of skills available and the needs of the labour market. This will require a very high level of training and retraining offers tailored to the professional profile of Ukrainians. Moreover, it will be necessary to employ additional measures to prevent threats such as exploitation in the workplace, bullying or sexual harassment as those may be expected considering the scale of the phenomenon and low bargaining power of war refugees.

Preventing conflicts that may occur between Ukrainians and Poles is also a very important challenge. Such a large influx of foreigners affecting the daily life of the host society has the potential to cause conflicts. Even though in the short term, due to the uniqueness of the situation, tensions can be easily avoided, they will certainly emerge in the medium and long term. Especially people using public services may experience a deterioration in the standard of living due to the presence of a significant number of war refugees, who will also be entitled to benefit from state support. A similar situation may also take place in the labour market, with possible adverse effects, particularly on the local scale. These risks should be identified, monitored and addressed through well-tailored public policies, including communication campaigns.

The issue of migration has for many years been included on the list of key political topics both nationally and internationally. The inflow or outflow of people to or from a given country is the axis of dispute in virtually every election campaign. Thus there is a risk that the presence of war refugees in Poland could easily become the subject of tense political debate, with all the accompanying negative consequences.
